# A glutamine ‘tug-of-war’: targets to manipulate glutamine metabolism for cancer immunotherapy

**DOI:** 10.1093/immadv/ltab010

**Published:** 2021-06-01

**Authors:** Laura J Pallett, Sarah Dimeloe, Linda V Sinclair, Adam J Byrne, Anna Schurich

**Affiliations:** 1 Division of Infection and Immunity, Institute of Immunity and Transplantation, University College London, London, UK; 2 Institute of Immunology and Immunotherapy, Institute of Metabolism and Systems Research, College of Medical and Dental Sciences, University of Birmingham, Birmingham, UK; 3 Division of Cell Signalling and Immunology, School of Life Sciences, University of Dundee, Dundee, UK; 4 Inflammation, Repair and Development Section, National Heart and Lung Institute, Imperial College London, London, UK; 5 Department of Infectious Diseases, School of Immunology and Microbial Sciences, King’s College London, London, UK

**Keywords:** glutamine, T cells, cancer immunotherapy

## Abstract

Within the tumour microenvironment (TME), there is a cellular ‘tug-of-war’ for glutamine, the most abundant amino acid in the body. This competition is most evident when considering the balance between a successful anti-tumour immune response and the uncontrolled growth of tumour cells that are addicted to glutamine. The differential effects of manipulating glutamine abundance in individual cell types is an area of intense research and debate. Here, we discuss some of the current strategies in development altering local glutamine availability focusing on inhibition of enzymes involved in the utilisation of glutamine and its uptake by cells in the TME. Further studies are urgently needed to complete our understanding of glutamine metabolism, to provide critical insights into the pathways that represent promising targets and for the development of novel therapeutic strategies for the treatment of advanced or drug resistant cancers.

## Introduction

Glutamine, the most abundant amino acid in the body, is a highly versatile precursor, contributing to several metabolic and biosynthetic pathways [1]. The discovery that cancer cells rely on glutamine (obtained from the local microenvironment, synthesised or generated by autophagy) to fuel growth, was made as early as 1955 [1–3]. Glutamine is not a classically essential amino acid, as it can be synthesised by glutamine synthase from glutamate and ammonia and some tumours have been shown to use autophagy to break down proteins to release amino acids including glutamine [4,5]. However, glutamine can be viewed as a conditionally essential amino acid for lymphocytes and many tumours, as these cells consume more glutamine than they can make and thus rely on its uptake from their environment [6,7]. This is of particular relevance, as lymphocytes, especially activated T cells, are in direct competition with tumour cells for this vital nutrient. Indeed, patients whose tumours display signatures associated with high glutamine metabolism, and thus potentially restricting glutamine availability to the immune system, have poor overall survival rates [8,9]. Therefore, the idea of manipulating tumour glutamine metabolism as a therapeutic strategy is an area of intense research. Whilst initial *in vitro* experimentation has been promising [10–12], systemic side effects in some early clinical trials have cautioned against reagents with broad activity [13–15]. Here, we review some of the advances made to date and the most exciting current strategies in clinical trials for oncology.

## Glutamine and its metabolites

**Figure 1.**
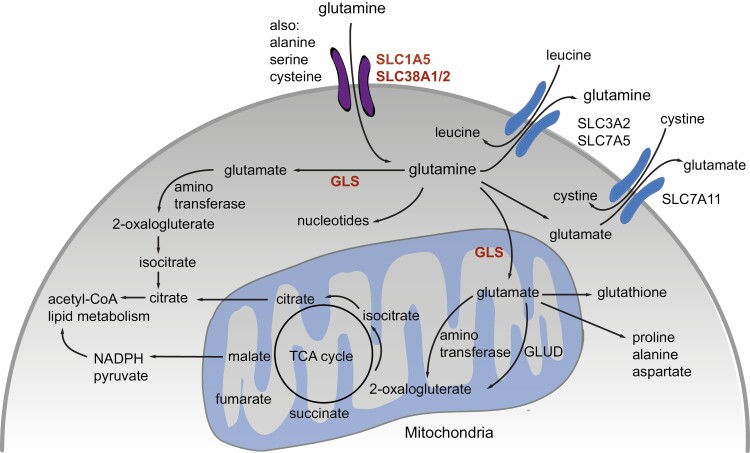
Glutamine is taken up into cells via glutamine transporters including SLC1A5 (ASCT2), SLC38A1 (SNAT1) and SLC38A2 (SNAT2). Inside the cell glutamine can serve as a substrate for various pathways, such as the synthesis of nucleotides. Glutamine is converted by cytosolic and mitochondrial glutaminases to glutamate a substrate for conversion by aminotransferase or glutamate dehydrogenase to 2-oxalogluterate, which enter the TCA cycle, for the generation of ATP, or the production of acetyl-CoA and NADPH, both of which can fuel lipid metabolism or generate pyruvate. Glutamate further contributes as a precursor to the generation of the anti-oxidant glutathione and amino acids proline, alanine and aspartate. Glutamate can be exported from the cell to allow uptake of cystine, through the antiporter SLC7A11 (xCT). Other essential amino acids, including methionine, leucine and tryptophan are brought into the cell through the anti-porter SLC7A5, which uses glutamine as the exchange currency.

Glutamine is largely obtained through the diet but can also be synthesised *de novo* through activity of glutamine synthase [1]. Under conditions of nutrient starvation, glutamine can also be acquired through the autophagic break-down of macromolecules [1,16]. Glutamine provides fuel for rapidly dividing cells, including tumour cells and lymphocytes and can become essential in these situations. These highly proliferative cells import or take up glutamine through cell surface transporters [17]. Many nutrient transporters are overexpressed by tumour cells. For example, the alanine–serine–cysteine transporter 2 (ASCT2), otherwise known as SLC1A5, and SLC38A2 (SNAT2), are the primary transporters responsible for glutamine uptake in cancer cells (Fig. 1) [2,17–20].

Glutamine is indispensable for many intracellular biosynthetic and metabolic processes including the synthesis of nucleotides, hexosamines, and other non-essential amino acids, maintaining redox balance, glycosylation, the production of extracellular matrix proteins and in epigenetic regulation [21–24]. To enable glutamine to fuel the tricarboxylic acid cycle (TCA), cells require the mitochondrial enzyme glutaminase (encoded by *GLS*), which catalyses the conversion of glutamine to glutamate. The subsequent conversion of glutamate to 2-oxalogluterate (α-ketoglutarate; α-KG), can be achieved by two distinct pathways, either via aminotransferases or glutamate dehydrogenases [1,2]. 2-oxalogluterate is then primarily converted to isocitrate and citrate in the TCA cycle. Citrate can either remain in the TCA cycle to form malate or be exported to generate acetyl-CoA to fuel lipid metabolism (Fig. 1). Malate can also be exported to generate pyruvate, coupled to the reduction of NADP^+^ to NADPH, the latter again fuelling lipid metabolism.

Additionally, glutamate can be exported from cells via the anti-porter SLC7A11 (xCT) in exchange for cystine (Fig. 1). Cystine can then be reduced in the cell to cysteine, which is either metabolised further or can again be released when glutamate is taken up. T cells express very low levels of SLC7A11 [25] (and ImmPRes accessed here: http://immpres.co.uk/). Furthermore, this transporter has been described to be non-functional in T cells [26]. In line with this, an approach to block SLC7A11 impairs glutamate/cystine exchange in tumour cells but with only a moderate influence on T cell function [27]. Moreover, we speculate that blockade of SLC7A11 activity might even enhance T cell function in the tumour, as T cells are partially dependent on extracellular cysteine, taken up via SLC1A5 (Fig. 1), to satisfy their demand. Only after activation do effector T cells express the enzyme cystathionase [25,28] which would allow them to make cysteine from methionine. Thus T cells directly compete with highly proliferative tumour cells for these metabolites, as well as for glutamine [29].

Glutamate is a signalling molecule in its own right, being a vital neurotransmitter in the central nervous system [26]. Interestingly, T cells express two distinct receptors for glutamate sensing; metabotropic glutamate receptor 5 (mGlu5R) which is constitutively expressed and involved in restraining pro-inflammatory cytokine release and proliferation in naive/resting T cells [30], and mGlu1R, which is only expressed upon activation of T cells. mGlu1R has been described as imparting an opposing and dominant effect over mGlu5R, thus supporting effector cytokine release, differentiation, and proliferation [26] and may play a role in the inhibition of activation induced cell death [31]. These findings therefore implicate glutamate (from the ongoing catabolism of glutamine) in the tumour microenvironment (TME) as a strong immunomodulator of T cell function.

Glutamine uptake is enhanced in T cells upon T cell receptor (TCR) stimulation and is critical for their survival, proliferation and effector function [32,33]. Increased expression of the glutamine transporters SLC1A5 (ASCT2), SLC38A1 (SNAT1), SLC38A2 (SNAT2) mediate elevated glutamine uptake in activated T cells [32,33] and expression of glutaminolytic enzymes is concomitantly increased [33]. These changes are driven by costimulation, for example, via CD28 signalling [33] and induction of Myc expression [10,34]. Glutamine supports T cell function in a number of ways, including facilitating the uptake (via direct exchange) of other amino acids including leucine, which is a key activator of the cellular metabolic regulator mechanistic target of rapamycin (mTOR) [16,32]. When T cells lack SLC1A5 or are cultured in glutamine-free media they fail to properly engage mTORC1 - providing evidence for the role of glutamine as a ‘signalling’ molecule [32,35].

Additionally, glutamine is used to fuel the TCA cycle, particularly in conditions where glucose availability might be limited, such as in the TME [36]. Importantly, distinct immune cell subsets demonstrate differential reliance on glutamine, and are therefore likely to be impacted in different ways by its depletion in the TME. Recently, using cutting edge radiolabelled glucose and glutamine *in vivo* uptake studies Reinfeld *et al.* show that in the TME (on a per cell basis) tumour cells are the biggest consumers of glutamine, followed by myeloid-derived suppressor cells (MDSC), with immune cells including tumour-associated macrophages and T cells in comparison only taking up comparatively small amounts [37]. Notably, in both mice and humans, glutamine deprivation favours the differentiation of regulatory T cells (T_REG_), which are immune-suppressive and highly proliferative *in vivo* [38–40]. More recently, it was shown that by inhibiting the conversion of glutamine to glutamate it is possible to influence memory CD4^+^ T differentiation, particularly altering the balance between Th1 and Th17 CD4^+^ T cells [41]. Furthermore, glutamine synthase inhibition also increases the accumulation of pro-inflammatory macrophages with the capacity to further enhance lymphocyte recruitment [42]. Therefore, targeting tumour cell glutamine metabolism may result in pleiotropic effects which can be exploited to target additional cells present in the tumour microenvironment, including immune cell populations.

Thus glutamine metabolism is emerging as an interesting target for cancer immunotherapy. Here, we will introduce three approaches being evaluated for immunotherapeutic intervention that harness the metabolic control of local glutamine levels to limit tumour cell growth and enhance anti-tumour T cell function. We will discuss the potential of each strategy in turn.

1) Inhibition of glutaminase (GLS) – a crucial step in the utilisation of glutamine

Many human cancers, including hepatocellular carcinoma, ovarian cancer, osteosarcoma, colorectal cancer, and breast cancer are characterised by increased GLS expression – significantly correlating with patient survival [43–46]. Similarly, elevated GLS expression, and its activity, is associated with high grade lesions and metastatic cancer [47]. In particular, using data curated by *The Cancer Genome Atlas,* Edwards *et al.* recently highlighted the inverse relationship between gene signatures associated with high levels of glutamine metabolism (based on expression of glutamine utilising enzymes and glutamine transporter expression) and anti-tumour T cell effector function in triple negative breast cancer. Similarly, overall survival rates are significantly worse in patients harbouring tumours that metabolise high levels of glutamine [8].

The close connection between tumour cell growth, glutamine utilisation, GLS expression, and the associated impact on T cell activation and effector function have led to targeting suppression of GLS with either small molecule inhibitors or genetic knockdown approaches. Promisingly, small molecule GLS inhibitors have been shown to exhibit anti-proliferative activity and reduce tumour burden [43,48,49] across a variety of tumours, including lymphoma, breast, pancreatic, non–small cell lung and renal cancers [9,10,48,50,51]. In line with these clinical observations, the effector function of anti-tumour T cells is markedly improved if tumour cells lose GLS activity *in vitro* using cutting-edge techniques such as CRISPR-Cas9, attributable to a reduction in tumour-cell-driven glutamine depletion and a concomitant increase in local glutamine availability to T cells [8].

Many potent small molecule inhibitors have been developed to target GLS disturbing further aspects of glutamine metabolism, including two compounds of note - bis-2-(5-phenylacetamido-1,3,4-thiadiazol-2-yl)ethyl sulfide (BPTES) and CB-839 [52]. Although BPTES showed initial promise *in vitro* inhibiting tumour cell proliferation, its advanced clinical development has been limited due to ongoing issues with bioavailability and poor drug solubility [53]. To improve solubility, a number of BPTES derivatives have been developed including the more potent, and selective inhibitor, CB-839 [49,54]. CB-839 has been reported to have broad antiproliferative activity against both solid tumours and haematological malignancies, including difficult to treat, triple-negative breast cancer and pancreatic ductal adenocarcinoma *in vitro* models [49,55,56]. The therapeutic addition of CB-839 led to a marked decrease in tumour cell glutamine consumption, glutamate production and several TCA intermediates. Importantly, numerous clinical trials using CB-839 as either a monotherapy or as a combination therapy are ongoing (detailed on the NIH website: https://www.cancer.gov/about-cancer/treatment/clinical-trials/intervention/glutaminase-inhibitor-cb-839), and time will tell the *in vivo* impact on immune cell function.

2) Broad targeting of glutamine metabolism

As introduced in the previous section, many of the initial efforts to target glutamine in oncology focused on the first step of glutaminolysis with specific GLS inhibitors [10]. Although these inhibitors demonstrated efficacy *in vitro*, it has become increasingly clear that GLS inhibition is much less effective *in vivo* [56]. Therefore, as an alternative to inhibiting just one enzymatic step, drugs that comprehensively manipulate glutamine metabolism more broadly such as the glutamine antagonist 6-diazo-5-oxo-L-norleucine (DON) that inhibits a range of glutamine-requiring enzymes including GLS have been developed [57]. Early studies over 50 years ago reported the ability of DON to potently inhibit tumour cell growth, and to simultaneously alter the metabolic landscape of the TME. However, the full clinical development of DON was abandoned after early phase I and phase II trials, due to unacceptable toxicity affecting the gastrointestinal tract [13–15].

More recently, the development of a series of pro-drugs based on DON, designed to mitigate overt toxicity, have offered hope for clinical translation. The most promising of which is a pro-drug JHU-083. JHU-083 markedly enhances endogenous anti-tumour immunity, with significant improvements in the overall survival of tumour-bearing mice [58]. Interestingly, Leone *et al.* have recently shown that treatment with JHU-083 increases the number of tumour-specific CD8^+^ T cells infiltrating the TME, these T cells are more proliferative, and appear to be robustly activated and less exhausted with improved effector functionality [58], in line with another finding that transient GLS-inhibition increases T-bet expression, skewing responses towards Th1 and increased cytotoxic T cell activity [41].

Importantly, the tumour specific CD8^+^ T cell compartment adapts to the metabolic environment of glutamine blockade by switching their ‘metabolic profile’, instead generating high levels of acetyl-CoA to fuel the TCA cycle by upregulating acetate metabolism rather than maintaining an over-reliance on glutaminolysis [58], while tumour cells largely failed to do the same.

Beyond the initial study by Leone *et al.* highlighting the differential ability of JHU-083 to disable tumour cells and enhance T cell function, Oh *et al.* further report that therapeutic targeting of glutamine metabolism with JHU-083 not only prevents tumour cell growth but dramatically changes the cellular composition of the TME. It seems that JHU-083 can prevent the generation, recruitment, and reprogramming of immunosuppressive myeloid cell populations, significantly reducing the number of suppressive MDSC entering the TME. Mechanistically JHU-083 induces either the local apoptosis of MDSC population and/or promotes their differentiation into mature, pro-inflammatory macrophages, with an improved capacity for antigen presentation [59].

Further support for the clinical development of pro-drugs such as JHU-083, comes from the ability of JHU-083 to prevent the development of metastatic disease in murine models. Treatment of tumour-bearing mice with JHU-083 showed an altered metabolic milieu at distinct metastatic sites as well as that within the primary tumour. Secondly, and perhaps more relevant to the current therapeutic landscape is the ability of JHU-083 to enhance the therapeutic efficacy of immune checkpoint blockade in immunotherapy-resistant tumours [59].

Taken together, these data provide a strong preclinical rationale for strategies antagonising glutamine metabolism as a means of enhancing immunotherapy for cancer.

3) Specific manipulation of glutamine transporter activity

There is little doubt that manipulating glutamine metabolism in the TME is an encouraging therapeutic strategy in the treatment of certain cancers, including triple negative breast cancer. In parallel, another strategy under consideration is the blockade of cellular glutamine uptake, which will also impact additional activities of glutamine beyond its metabolism, including facilitation of the uptake of other amino acid transporters that require glutamine antiport (for example via, SLC7A5; LAT1, Fig. 1). Three years ago, Schulte *et al.* first demonstrated the use of the glutamine transporter inhibitor, V-9302 a potent small molecule antagonist designed to target SLC1A5/ASCT2 [60]. Following just a single dose of V-9302, glutamine uptake into malignant cells of mice bearing tumour HCC-1806 cell-line xenografts reduced by up to 50%, attenuating tumour cell growth and proliferation [60]. Importantly, this study raised the possibility that the therapeutic benefit of V-9302 was tumour-cell specific, and its use *in vivo* may ‘protect’ T cell immune surveillance.

More recently understanding the utility of V-9302 in oncology has been taken a step further. A study by Edwards *et al.* has shown that while limiting the growth of orthotopic E0771 tumours, V-9302 increased the infiltration of CD8^+^ T cells into the TME, that were more activated and exhibited improved cytolytic and non-cytolytic effector function. Additionally, treatment with V-9302 concomitantly altered the predominance of CD4^+^ T cell subsets, increasing the number of Th1 cells producing the anti-tumour effector molecule IFNγ ^+^ in the TME. Key to the metabolic flexibility of these anti-tumour T cells, was their ability to adapt, with the compensatory upregulation of alternative glutamine transporters such as SLC6A14, that was not seen on the tumour cells, allowing for superior anti-tumour T cell responses, while curtailing tumour cell growth [8].

Taken together, the tumour-selective blockade of glutamine uptake represents a promising approach to combat cancer, providing a two-pronged attack, boosting anti-tumour immune responses while crippling tumour cell metabolism.

## Combining glutamine manipulation with check-point blockade

Recent findings have shown that limiting glutamine availability can lead to increased expression of PD-L1 on tumour cells [61,62] and a concomitant increase in expression of the death ligand Fas on T cells. Furthermore, ligation of both checkpoint inhibitors PD-1 and CTLA-4 significantly impairs activation-induced upregulation of glutamine transporters and glutamine uptake in T cells [61] consistent with their antagonism of CD28 signalling, a key driver of glutamine metabolism in these cells. Thus, while increased PD-L1 expression on tumour cells can suppress the T cell response, it also makes cancer cells amenable to checkpoint blockade therapy with PD-1/PD-L1 inhibition promoting tumour cell susceptibility to T cell mediated killing [61,63].

## Detection of tumours amenable to glutamine manipulation

It is likely that the above discussed therapies will benefit some patients more than others, due to individual differences in glutamine metabolism and tumour heterogeneity. To allow better prediction of therapeutic success, before starting therapy, glutamine addicted tumours could be revealed by positron emission tomography (PET) scans making use of glutamine tracers, a strategy currently being tested [64,65]. Soon this might allow us to establish not only how glutamine is distributed, both systemically and within the TME, but also how effectively a specific drug is decreasing glutamine uptake by cancer cells. This imaging strategy is currently being researched using PET tracers (for example using 11C-glutamine [65]) to identify cancerous tumours by detecting any increases in the glutamine metabolism rate, which are predicted to be higher compared with that of normal, healthy cells in the body. The Vanderbilt Center for Molecular Probes is hosting five clinical trials designed to test the effectiveness of tracers including 11C-Glutamine and 18F-FSPG, a new radiopharmaceutical used in PET scans, tracing various types of tumours, including lung, liver, ovary, and colon cancer. As introduced earlier, *in vivo* use of radiolabelled glucose and glutamine has revealed that tumour cells are the greatest consumers of glutamine, while MDSC are the greatest consumers of glucose (on a cell per cell basis) [37]. Of note, tumour cells outnumber all other cells in the TME and therefore represent the net major consumers of both nutrients [37]. Tumour infiltrating T cells appear to take up broadly equivalent amounts of glucose and glutamine; this may indicate plasticity or a retained capacity to balance metabolic pathways, as has been demonstrated in previous *in vivo* labelling infection studies [36,66]. Altogether, these studies support the notion that inhibiting glutamine uptake or metabolism would preferentially target tumour cells and associated suppressor cells.

Tracing glutamine uptake could offer additional advantages, as some cancers, such as gliomas in the brain, are difficult to detect using established glucose tracing, since the healthy brain tissue itself is a major consumer of glucose thereby effectively hiding the malignant tissue; use of glutamine tracing here demonstrated experimental success [67].

## Future outlook

Although targeting glutamine metabolism represents a promising strategy for the clinical design of therapeutic agents, several challenges remain. Since glutamine is essential for cellular proliferation, function and ultimately, host defence, there is the potential for unwanted side effects by targeting glutamine or associated processes. Additionally, some cancers exhibit glutamine independence and therefore resistance to therapies that restrict glutamine metabolism. For example, high expression of GLS can drive glutamine synthesis from glutamate, maintaining cell proliferation during glutamine deprivation [68]. It is therefore becoming increasingly apparent that future therapeutic strategies should be devised in the context of the metabolically hostile TME. For example, glutamine deficiency may be a critical barrier to using CAR T cell therapy for treating solid tumours; pre-adaptation of CAR T cells in conditions which reproduce the *in vivo* metabolic environment of tumours may improve anti-tumour responses *in vivo*. Indeed, recent evidence suggests that pre-adaptation to glutamine deprivation *in vitro* enhances CD8^+^ T cell responses when later adoptively transferred *in vivo*, although the mechanisms behind this are not yet completely understood [41,69,70].

One further complexity is the potential for epigenetic modulation in response to the TME. Metabolism and epigenetic regulation of T cells are heavily intertwined; epigenetic modifiers utilise products of key cellular metabolic processes as either cofactors or substrates, and regulation of the epigenetic landscape can directly influence cellular metabolism [71,72]. In particular, disrupted glutamine metabolism results in depletion of α-KG ^4^, which in turn influences the epigenetic landscape of CD8^+^ T cells in the tumour microenvironment. For example, succinyl-CoA derived from α-KG oxidation provides substrate for histone succinylation [73], a process which is tightly linked to tumour cell proliferation. In addition glutamine-derived α-KG is crucial for the activity of histone and DNA demethylation enzymes such as Jumonji N/C-terminal domain (JmjC) and ten-eleven translocation (TET) enzymes [74], which are necessary for anti-tumour immunity. Although this interplay between glutamine metabolism, epigenetic regulation and the tumour microenvironment remains to be fully elucidated, combined epigenetic-directed and metabolic therapies might hold potential to improve current cancer therapies such as checkpoint blockade. In line with this, a recent study demonstrated the potential for dietary intervention, showing that glutamine supplementation, rather than the nutrient-limiting approaches discussed here, had the potential to limit tumour cell growth by suppressing epigenetically activated oncogenic pathways [75].

In conclusion, further studies in anticipation of a complete and robust understanding of glutamine metabolism in tumour and immune cells within the TME context is of the utmost importance, as it provides valuable insights into the pathways that could be targeted for the development of novel therapeutic strategies for the treatment of advanced or drug resistant cancers.

## Abbreviations

ASCT2: alanine–serine–cysteine transporter 2
DON: 6-diazo-5-oxo-L-norleucine
α-KG: α-ketoglutarate
MDSC: myeloid-derived suppressor cells
mGluXR: metabotropic glutamate receptor X
mTOR: metabolic regulator mechanistic target of rapamycin
PET: positron emission tomography
TCA: tricarboxylic acid cycle
TME: tumour microenvironment
T_REG_: regulatory T cells
